# Analysis of candidate genes for age-related macular degeneration subtypes in the Japanese population

**Published:** 2011-10-22

**Authors:** Koji Tanaka, Tomohiro Nakayama, Mitsuko Yuzawa, Zhaoxia Wang, Akiyuki Kawamura, Ryusaburou Mori, Hiroyuki Nakashizuka, Naoyuki Sato, Yoshihiro Mizutani

**Affiliations:** 1 Department of Ophthalmology, Nihon University School of Medicine, Tokyo, Japan; 2 Division of Laboratory Medicine, Department of Pathology and Microbiology, Nihon University School of Medicine, Tokyo, Japan

## Abstract

**Purpose:**

Age-related macular degeneration (AMD) is thought to be a polygenetic disease. It is divided into three subtypes; neovascular AMD (nAMD), polypoidal choroidal vasculopathy, and retinal angiomatous proliferation (RAP). These subtypes are thought to have different pathophysiological and genetic backgrounds. We aimed to investigate the relationships between single nucleotide polymorphisms (SNPs) in candidate genes and subtypes of AMD in the Japanese population.

**Methods:**

We genotyped 685 AMD patients and 277 controls for four SNPs of the selected candidate genes: rs800292 in complement factor H, rs10490924 in age-related maculopathy susceptibility 2 (*ARMS2*), rs2301995 in elastin (*ELN*), and rs1801133 in methylenetetrahydrofolate reductase (*MTHFR*). Case-control studies were performed using these AMD subtypes. Logistic regression analysis was performed using a history of hypertension, diabetes mellitus, and smoking as cardiovascular risks.

**Results:**

The genotype-dominant or recessive distribution of all four SNPs differed significantly between the controls and the AMD patients. In the subtype analysis, there were significant differences between the controls and the AMD patients in genotype distributions. This was true for all AMD subtype analyses of both rs800292 (complement factor H) and rs10490924 (*ARMS2*). Logistic regression analysis indicated the TT genotype of the *ARMS2* gene to be significantly more common in RAP patients (p=1.54×10^−13^, odds ratio: 22.18). In contrast, there were significant differences in the genotype distribution between the controls and nAMD patients only for rs2301995 (*ELN*, p=0.022) and rs1801133 (*MTHFR*, p=2.50×10^−3^).

**Conclusions:**

Our results indicate that SNPs of the *ARMS2* gene may serve as strong genetic markers of RAP, and that SNPs of the *ELN* and *MTHFR* genes are potential genetic markers for nAMD.

## Introduction

Age-related macular degeneration (AMD) is a leading cause of blindness in Western countries, and its prevalence is increasing in Japan [1,2]. AMD is thought to be a heterogeneous multifactorial disease associated with several environmental factors and genetic variants. Factors such as hypertension [3] and cigarette smoking [4] are closely related to the development of AMD. Identification of AMD-susceptibility genes might increase our ability to predict the risk of developing AMD. Complement factor H (*CFH*) is reportedly related to AMD in Caucasians [5]. In addition, age-related maculopathy susceptibility 2 (*ARMS2*) and high-temperature requirement factor A1 have been shown to be associated with AMD in both Japanese and Caucasian patients [6-10]. The *CFH* polymorphism Y402H has been reported to be associated with AMD in studies conducted worldwide. However, several Japanese studies have found Y402H to be unrelated to AMD [11-14]. Other studies of the Japanese population have shown an association between *CFH* rs800292 and AMD [14-16]. Two studies examined gene polymorphisms associated with retinal angiomatous proliferation (RAP) [17,18].

In Japan, exudative AMD includes both neovascular AMD (nAMD) and serous retinal pigment epithelial detachment without choroidal neovascularization, while polypoidal choroidal vasculopathy (PCV) and RAP have been defined as specific forms of exudative AMD [19]. Reported frequencies of each subtype in the Japanese population are 35.3% for nAMD, 54.7% for PCV, and 4.5% for RAP [20]. PCV occurs in less than 8% of Caucasians [21-24]. Histopathological examination of PCV demonstrated choroidal vessels to be similar to those in atherosclerosis or choroidal neovascularization [25]. As these AMD subtypes may have different pathophysiological and genetic backgrounds, we undertook this study of the associations between genetic markers or mutations and individual AMD subtypes.

Several candidate genes for atherosclerosis have been reported. Elastin (*ELN*) is among the markers of atherosclerosis [26] and neovascularization [27,28]. Kondo et al. [29] reported an *ELN* gene polymorphism (rs2301995) to confer susceptibility only for PCV, not for nAMD. They reported the frequency of the rs2301995 polymorphism in the *ELN* gene to differ significantly between the PCV and control groups.

Methylenetetrahydrofolate reductase (MTHFR) is a key enzyme in the homocysteine cycle. Hyperhomocysteinemia reportedly is a risk for AMD [30,31] and induces vascular endothelial growth factor in the retina of a rat model [32]. Thus, the *MTHFR* gene is among the candidates for atherosclerosis susceptibility genes [33,34].

The present study aimed to use single-nucleotide polymorphisms (SNPs) to investigate the relationships between AMD subtypes and potential susceptibility genes, *ARMS2*, *CFH*, *MTHFR*, and *ELN*, in Japanese subjects. We determined the frequencies of three AMD subtypes, along with the age and gender distributions of the study’s participants. We also analyzed four SNPs for AMD subtype associations and calculated corresponding odds ratios (ORs).

## Methods

### Subjects

All AMD patients and control subjects were recruited at Nihon University Surugadai Hospital in Tokyo between 2001 and 2010. In total, 685 patients (478 men and 207 women; mean age 72.0±8.8 years) were diagnosed with AMD by color fundus photography, fluorescein angiography, and indocyanine green angiography. The patient group included 253 diagnosed with nAMD, 381 with PCV, and 51 with RAP. All study subjects were asked if they had a history of hypertension, diabetes mellitus, and/or smoking (including both previous and current smoking at the time of the study). In total, 277 subjects without AMD (111 men and 166 women; mean age 72.9±8.7 years) served as control subjects. There were no remarkable findings from the fundus examinations of the controls. Smokers were defined as current or former smokers, nonsmokers as subjects with no previous or current smoking history. Informed consent was obtained from all participants as per the protocol approved by the Human Studies Committee of Nihon University.

### Genotyping

DNA was extracted from peripheral blood leukocytes by the phenol and chloroform extraction technique [35,36]. Briefly, the samples were centrifuged for 10 min at 1,500× g, and the pellet was resuspended in 3 ml of solution 1, which consisted of 10 mM tris (pH 7.5), 10 mM KCl, 10 mM MgCl_2_, and Nonidet P40 (Sigma, St. Louis, MO), and incubated on a rotator for 30 min. The tube was centrifuged as described above for 10 min, and the supernatant was removed to another tube. Two milliliters of solution 2 (10 mM Tris (pH 7.5), 10 mM KCl, 10 mM MgCl_2_, 50 mM NaCl, 5 mM EDTA (pH 8.0), 25 ml 10% SDS) and 10.4 ml of proteinase K (20 mg/ml H_2_O; Sigma) were added to the supernatant. After incubation on a rotator at 37 °C overnight, 2.2 ml of phenol was added and incubate on a rotator for 30 min. The sample was centrifuged as described above for 10 min, and the supernatant was removed with a large-bore tip to another tube. Phenol purification was repeated twice: 2.2 ml of phenol/ chloroform/isoamyl alcohol was added, and incubation and centrifugation were performed described; 2.2 ml of chloroform was added and incubation and centrifugation were performed again. The 400 ml of aqueous layer was transferred carefully to a new 1.5-ml microcentrifuge tube to which 1 ml of cold 100% ethanol was added, mixed, and incubated for 15 min at 20 °C. Genotyping was performed using the TaqMan SNP Genotyping Assay (Applied Biosystems, Carlsbad, CA). TaqMan SNP Genotyping Assays were performed using the method for Taq amplification [37,38].

Plates were read on an SDS 7700 instrument with the end-point analysis mode of the SDS, version 1.6.3, software package (Applied Biosystems). Genotypes were determined visually, based on the dye-component fluorescent emission data depicted in the X-Y scatter plot of the SDS software. Genotypes were also automatically determined by applying signal processing algorithms of the software [35,36].

### Statistical analysis

Data are shown as means, plus or minus standard deviation (±SD). Differences among the nAMD, PCV, RAP, and control groups were assessed by the ANOVA (ANOVA), followed by Fisher’s protected least significant difference (PLSD) test. Hardy–Weinberg equilibrium was assessed by chi-square analysis. The overall distribution of the SNP alleles was determined using 2×2 contingency tables. The distributions of SNP genotypes in the AMD patients and the controls were tested using a 2-sided Fisher’s exact test and multiple logistic regression analysis. Statistical significance was established at p<0.05. Because we examined four SNPs, we applied multiple comparisons with a strict p value (0.05/SNP number in this study: four, p<0.0125).

Logistic regression analyses for assessing the contributions of major risk factors and the independence of each SNP in relation to AMD were performed using SPSS software for Windows, version 12 (SPSS, Chicago, IL).

## Results

The clinical features of AMD patients and the control group are shown in Table 1. There were no significant differences in age or history of hypertension between the AMD patients and the control group. There were significantly more males, and the frequencies of smoking were higher in the patient group than in the control group. However, there were significantly fewer subjects with diabetes mellitus in the patient group than in the control group.

**Table 1:** Characteristics of study participants.

**Parameters**	**Total AMD**		**Case**			**Control**
		**p-value**	**nAMD**	**PCV**	**RAP**	
No. of subjects	685		253	381	51	277
Age (mean±SD)	72.0±8.8	0.146	73.7±7.5	69.9±9.1	80.1±6.8	72.9±8.7
Male/female	478/207	<0.0001*	188/65	271/110	19/32	111/166
Hypertension (%)	39.1	0.254	38.3	39.4	41.2	43.0
Diabetes mellitus (%)	10.7	<0.0001*	12.6	9.4	9.8	19.5
Smoking (%)	34.7	<0.0001*	34.8	37.3	15.7	17.7

Abbreviations: AMD represents age-related macular degeneration, nAMD represents neovascular age related macular degeneration, PCV represents polypoidal choroidal vasculopathy, RAP represents retinal angiomatous proliferation, N represents number, SD represents standard deviation. Information on hypertension, diabetes mellitus and smoking was obtained from the medical history of each patient. p-values reflect comparisons between case and control groups. p-values were calculated using Fisher's exact test. *p<0.05

Genotype and allele distributions of the four SNPs are shown in Table 2. All four SNPs in the controls were in Hardy–Weinberg equilibrium (data not shown, p>0.05). The genotype-dominant or recessive distribution of all four SNPs differed significantly between the control and AMD groups. Subtype analysis revealed a significant difference between the controls and the AMD patients in genotype distributions for all AMD subtypes, and both rs800292 (*CFH*) and rs10490924 (*ARMS2*). There were significant differences in the genotype distributions of rs2301995 (*ELN*) and rs1801133 (*MTHFR*) between the controls and nAMD patients.

**Table 2:** Genotype and allele distributions in AMD patients and control group

**Susceptibility genotype**	**Total AMD patients**			**nAMD**			**PCV**			**RAP**			**Control**	
	**number**	**%**	**p value**	**number**	**%**	**p value**	**number**	**%**	**p-value**	**number**	**%**	**p-value**	**number**	**%**
rs800292** (*CFH*) Genotype**														
G/G	386	56.4	p<0.0001*	147	58.1	p<0.0001*	210	55.1	p<0.0001*	29	56.9	0.011*	100	36.1
G/A	263	38.4		93	36.8		150	39.4		20	39.2		141	50.9
A/A	36	5.3		13	5.1		21	5.5		2	3.9		36	1.3
**G/G**	386	56.4	p<0.0001*	147	58.1	p<0.0001*	210	55.1	p<0.0001*	29	56.9	0.005*	100	36.1
**GA/AA**	299	43.6		106	41.9		171	44.9		22	43.1		177	63.9
***AA***	36	5.3	p<0.0001*	13	5.1	0.002*	21	5.5	0.0008*	2	3.9	0.063	36	1.3
***GA/GG***	649	94.7		240	94.9		360	94.5		49	96.1		241	8.7
rs800292** (*CFH*) Allele**														
G	1035	75.5	p<0.0001*	387	76.5	p<0.0001*	570	74.8	p<0.0001*	78	76.5	0.004*	341	61.6
A	335	24.5		119	23.5		192	25.2		24	23.5		213	38.4
rs10490924** (*ARMS2*) Genotype**														
G/G	142	20.7	p<0.0001*	46	18.2	p<0.0001*	94	24.7	p<0.0001*	2	3.9	p<0.0001*	99	35.7
G/T	253	36.9		81	32.0		162	42.5		10	19.6		142	51.3
T/T	290	42.3		126	49.8		125	32.8		39	76.5		36	1.3
**GG**	142	20.7	p<0.0001*	46	18.2	p<0.0001*	94	24.7	0.002*	2	3.9	p<0.0001*	99	35.7
**GT+TT**	543	79.3		207	81.8		287	0.753		49	0.961		178	64.3
***TT***	290	42.3	p<0.0001*	126	49.8	p<0.0001*	125	32.8	p<0.0001*	39	76.5	p<0.0001*	36	13.0
***GT+GG***	395	57.7		127	50.2		256	67.2		12	23.5		241	87.0
rs10490924** (*ARMS2*) Allele**														
G	537	39.2	p<0.0001*	173	34.2	p<0.0001*	350	45.9	p<0.0001*	14	13.7	p<0.0001*	340	61.4
T	833	60.8		333	65.8		412	54.1		88	86.3		214	38.6
rs2301995** (*ELN*) Genotype**														
C/C	457	66.7	0.037*	181	71.5	0.013*	239	62.7	0.237	37	72.5	0.176	165	59.6
C/T	207	30.2		64	25.3		130	34.1		13	25.5		96	34.7
T/T	21	3.1		8	3.2		12	3.1		1	2		16	5.7
**CC**	457	66.7	0.035*	181	71.5	0.004*	239	62.7	0.41	37	72.5	0.08	165	59.6
**CT+TT**	228	33.3		72	28.5		142	37.3		14	27.5		112	40.4
***TT***	21	3.1	0.047*	8	3.2	0.148	12	3.1	0.099	1	2	0.259	16	5.8
***CT+CC***	664	96.9		245	96.8		369	96.9		50	9.8		261	94.2
rs2301995** (*ELN*) Allele**														
C	1121	81.8	0.013*	426	84.2	0.003*	608	79.8	0.206	87	85.3	0.059	426	76.9
T	249	18.2		80	15.8		154	20.2		15	14.7		128	23.1
rs1801133** (*MTHFR*) Genotype**														
C/C	250	36.5	0.089	110	43.5	0.002*	123	32.3	0.692	17	33.3	0.819	81	29.2
C/T	330	48.2		105	41.5		198	52.0		27	52.9		152	54.9
T/T	105	15.3		38	15.0		60	15.7		7	13.7		44	15.9
**CC**	250	36.5	0.032*	110	43.5	0.001*	123	32.3	0.404	17	33.3	0.558	81	29.2
**CT+TT**	435	63.5		143	56.5		258	67.7		34	66.7		196	70.8
***TT***	105	15.3	0.829	38	15.0	0.783	60	15.7	0.962	7	13.7	0.696	44	15.9
***CT+CC***	580	84.7		215	85.0		321	84.3		44	86.3		233	84.1
rs1801133** (*MTHFR*) Allele**														
C	830	60.6	0.114	325	64.2	0.012*	444	58.3	0.564	61	59.8	0.558	314	56.7
T	540	39.4		181	35.8		318	41.7		41	40.2		240	43.3

Abbreviations: AMD represents age-related macular degeneration nAMD represents neovascular age-related macular degeneration PCV represents polypoidal choroidal vasculopathy RAP represents retinal angiomatous proliferation. p-values for the comparison between cases and controls. p-values for genotypes were calculated by Fisher's exact test. *p<0.05. p-values for multiple comparison. Bold font indicate dominant model and bold italic font indicate recessive models.

The results of logistic regression analysis, adjusted for confounding factors, are shown in Table 3. This analysis was performed for the dominant or recessive genotype models that showed significant results, as presented in Table 2. Susceptibility genotypes were those with high frequencies in patient groups in case-control studies. *CFH* genotype distributions differed significantly between the controls and all AMD subtypes, especially for nAMD (p=7.66×10^−8^; OR: 2.90). Similarly, the *ARMS2* genotype distributions differed significantly between the controls and all AMD subtypes. In particular, the TT genotype of the *ARMS2* gene was significantly more frequent in RAP patients (p=1.54×10^−13^, OR: 22.18). In contrast, SNPs in the *ELN* (p=0.022) and *MTHFR* (p=2.50×10^−3^) genes were associated only with nAMD, that is, not with either PCV or RAP.

**Table 3:** Logistic regression analysis with adjustment for confounding factors.

**Susceptibility genotype**	**Total AMD patients**			**nAMD**			**PCV**			**RAP**		
** **	**p-value**	**OR**	**95%CI**	**p-value**	**OR**	**95%CI**	**p-value**	**OR**	**95%CI**	**p-value**	**OR**	**95%CI**
rs800292** (*CFH*)**												
GG (rec)	2.36×10^−8^	**2.47**	1.79–3.41	7.66×10^−8^	**2.90**	1.96–4.27	1.42×10^−5^	**2.26**	1.55–3.27	0.029	**2.09**	1.08–4.06
GG+GA (dom)	3.33×10^−4^	**2.71**	1.57–4.67	6.22×10^−3^	**2.68**	1.32–5.34	8.46×10^−3^	**2.32**	1.23–4.39			
rs10490924** (*ARMS2*)**												
GT+TT (dom)	3.85×10^−6^	**2.29**	1.61–3.26	2.00×10^−5^	**2.59**	1.67–4.02	5.88×10^−3^	**1.75**	1.17–2.62	0.001	**11.60**	2.69–50.03
TT (rec)	2.37×10^−19^	**5.74**	3.76–8.77	3.24×10^−16^	**7.03**	4.40–11.23	1.54×10^−6^	**3.37**	2.09–5.42	1.54×10^−13^	**22.18**	9.74–50.50
rs2301995** (*ELN*)**												
CC (rec)				2.2×10^−2^	**1.58**	1.07–2.35						
CC+CT (dom)												
rs1801133** (*MTHFR*)**												
CC (rec)				2.50×10^−3^	**1.82**	1.23–2.68						
CC+CT (dom)												

Logistic regression analysis was performed for each genotype with adjustment for confounding factors (age, gender, hypertension, diabetes mellitus and smoking). Abbreviations: AMD represents age-related macular degeneration, nAMD represents neovascular age-related macular degeneration, PCV represents polypoidal choroidal vasculopathy, RAP represents retinal angiomatous proliferation, CI represents confidence intervals, dom represents dominant model, rec represents recessive model. p-values for comparisons between the case and control groups. p-values for genotypes were calculated using Fisher's exact test. p<0.05. Blanks indicate no siginificant difference.

Multiple logistic regression analysis was performed to determine the independence of the four SNPs as risk factors for AMD (Table 4). Each p value and 95% CI for the SNPs showed a statistically significant difference after adjustment for all confounding factors. Each SNP was confirmed to be an independent risk factor for AMD.

**Table 4:** Odds ratios and 95% confidence intervals for each model.

**Susceptibility genotype**	**p-value**	**OR**	**95% CI**
CFH recessive model	4.27×10^−7^	**2.17**	1.60–2.94
ARMS2 recessive model	2.76×10^−15^	**4.73**	3.22–6.99
ELN recessive model	0.018	**1.44**	1.06–1.96
MTHFR recessive model	0.037	**1.4**	1.02–1.94

p-values reflect the comparison between each gene. Abbreviations: OR represents odds ratio CI represents confidence intervals.

## Discussion

Our results indicated significant differences between all AMD subtypes and the controls in the distributions of genotypes and alleles of SNPs in the *CFH* and *ARMS2* genes. Both the *CFH* SNP (rs800292) and *ARMS2* SNP (rs10490924) are located within exons and are known to be I62V and A69S missense mutations. Although several studies have examined the functions of these genes, controversies concerning their exact functions persist [37,38].

As in previous reports, the SNPs of *CFH* (rs800292) and *ARMS2* (rs10490924) were both strongly associated with AMD in our study. In comparing these two genes, the *ARMS2* SNP is a more powerful marker of AMD than is the *CFH* SNP. The odds ratio (OR) for *CFH* was equal in the three subtypes, suggesting that *CFH* might be a marker for all AMD subtypes. In contrast, the OR for *ARMS2* was markedly higher for RAP than for the other subtypes. These results may support the theory suggested by Hayashi et al. [18]. They reported an SNP in the *ARMS2* gene to be highly associated with all three subtypes, with the association being strongest for RAP and weakest for PCV. They concluded that PCV and RAP might thus be subtypes of AMD that are genetically distinct from nAMD. We agree with their conclusion. *ARMS2* gene might serve as strong genetic markers of RAP. There may be pathological distinctions among these three subtypes. The significant differences between these two SNPs in case-control studies using AMD subtypes might be explained as follows: 1) The *CFH* and *ARMS2* genes may influence a common factor such aging in all three subtypes. 2) An unknown gene might affect each of these genotypes.

We examined the histopathology of human PCV specimens excised during the diagnosis of nAMD. CD68-positive cells were detected around hyalinized vessels. Hypoxia-inducible factor (HIF)-1alpha positive inflammatory cells were located in the stroma of these specimens. Our results indicate that the hyalinization of choroidal vessels, like arteriosclerosis, is characteristic of PCV [27]. Other investigators reported that *ELN* is highly associated with the calcification of elastic fibers in human atherosclerotic plaques [26]. Therefore, the *ELN* gene was considered to be a candidate gene for PCV. Because the rs2301995 SNP in the *ELN* gene was already reported to be associated with PCV [30], we conducted association studies for all three subtypes using this SNP. Since rs2301995 is located in the intron, it is not clear whether this SNP affects gene function. Although a previous study demonstrated TT homozygosity to be a risk factor for PCV, we found CC homozygosity to be higher in the nAMD group. These findings suggest that the CC homozygosity of rs2301995 is related to nAMD rather than PCV.

In addition to these results, several other reports have demonstrated a relationship between the ELN protein and neovascularization [27,28]. Skeie et al. [27] reported that *ELN* abnormalities might play a role in the neovascularization that occurs in AMD. Sivaprasad et al. [28] reported that serum ELN-derived peptides might increase the risk of conversion from early AMD to neovascular AMD. Taken together, these reports suggest that *ELN* might be related to neovascularization. To our knowledge, ours is the first report showing the *ELN* SNP (rs2301995) to be associated with nAMD but to have no association with PCV. The previously reported, opposite results may be due to the different sample sizes in our study and that of Kondo et al. [29], who analyzed only 74 cases. We believe our results to be more reliable than theirs because our sample was much larger. Since we investigated only one SNP, we cannot conclude that the *ELN* gene has no association with PCV.

It is known that rs1801133 is a missense mutation in the *MTHFR* gene, which is located in the exon known as A222V. Although Haas et al. [39] concluded that this variant was not related to AMD, there is a widely accepted view that homocysteine is a risk factor for atherosclerosis [31,33] and AMD [30]. Therefore, we examined the association between this variant and AMD subtypes in Japanese subjects. Significant differences were noted among the nAMD and control groups, suggesting *MTHFR* to be a potential marker of AMD. In addition, our results also indicate that nAMD might not be related to atherosclerosis. Most previous studies showed TT homozygosity to be more common in subjects with atherosclerosis than in those without it. However, we found the rate of CC homozygotes to be higher in the AMD group than in the controls. This is quite the opposite of previous reports on atherosclerosis. This variant may be a genetic marker that does not affect gene function, and the C or T allele may be linked to different susceptibility alleles for either atherosclerosis or AMD.

To our knowledge, this is the first application of logistic regression analysis to confounding factors known to be associated with AMD risk in three AMD subtypes (Table 3). All genotypes showing significant differences in the simple case-control study also yielded significant p values from the logistic regression analyses. These results suggest that each of the genotypes examined is an independent risk factor for AMD.

The present study is the first to examine potential correlations of the *MTHFR* and *ELN* genes with each of three AMD subtypes based on examinations of SNPs. Our findings suggest that polymorphisms of each gene are potentially useful genetic markers of AMD, and indicate that SNPs of the *ARMS2* gene may serve as strong genetic markers of RAP. The major limitation of this study is that only one SNP in each of the four genes was examined in a rather small numbers of patients. These results are preliminary, especially for RAP. Based on the present results, we plan further studies to investigate specific SNPs for each subtype of AMD, particularly those for PCV and RAP. Findings from these studies are anticipated to facilitate clarification of the causes of AMD.
